# Psammaplin A and Its Analogs Attenuate Oxidative Stress in Neuronal Cells through Peroxisome Proliferator-Activated Receptor γ Activation

**DOI:** 10.1021/acs.jnatprod.4c00153

**Published:** 2024-04-18

**Authors:** Rebeca Alvariño, Amparo Alfonso, Jioji N. Tabudravu, Jesús González-Jartín, Khalid S. Al Maqbali, Marwa Elhariry, Mercedes R. Vieytes, Luis M. Botana

**Affiliations:** † Departamento de Fisiología, Facultad de Veterinaria, IDIS, Universidad de Santiago de Compostela, Lugo 27002, España; ‡ Departamento de Farmacología, Facultad de Veterinaria, IDIS, Universidad de Santiago de Compostela, Lugo 27002, España; § School of Pharmacy and Biomedical Sciences, University of Central Lancashire, Preston, Lancashire PR1 2HE, United Kingdom

## Abstract

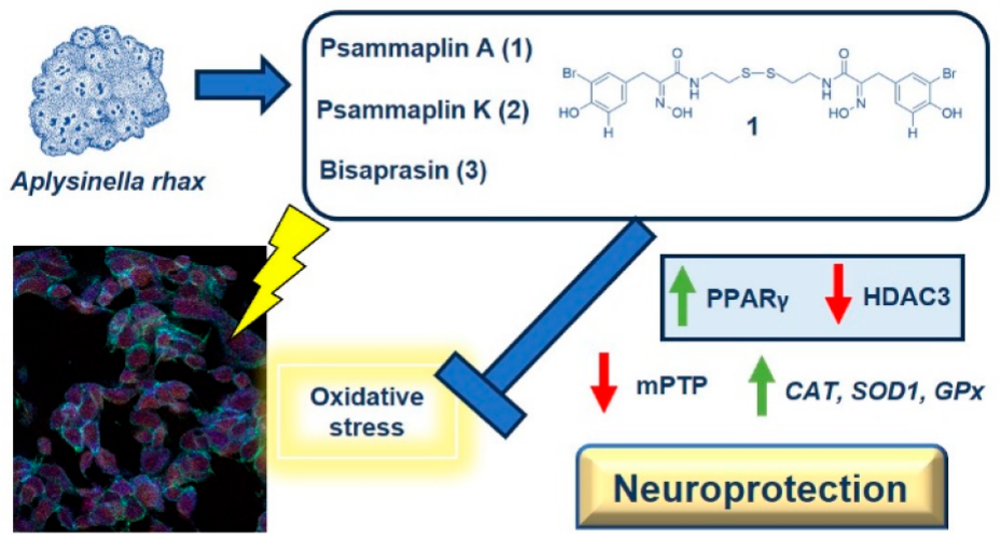

Psammaplins are sulfur containing bromotyrosine alkaloids that have shown antitumor activity through the inhibition of class I histone deacetylases (HDACs). The cytotoxic properties of psammaplin A (**1**), the parent compound, are related to peroxisome proliferator-activated receptor γ (PPARγ) activation, but the mechanism of action of its analogs psammaplin K (**2**) and bisaprasin (**3**) has not been elucidated. In this study, the protective effects against oxidative stress of compounds **1**–**3**, isolated from the sponge *Aplysinella rhax*, were evaluated in SH-SY5Y cells. The compounds improved cell survival, recovered glutathione (GSH) content, and reduced reactive oxygen species (ROS) release at nanomolar concentrations. Psammaplins restored mitochondrial membrane potential by blocking mitochondrial permeability transition pore opening and reducing cyclophilin D expression. This effect was mediated by the capacity of **1**–**3** to activate PPARγ, enhancing gene expression of the antioxidant enzymes catalase, nuclear factor E2-related factor 2 (Nrf2), and glutathione peroxidase. Finally, HDAC3 activity was reduced by **1**–**3** under oxidative stress conditions. This work is the first description of the neuroprotective activity of **1** at low concentrations and the mechanism of action of **2** and **3**. Moreover, it links for the first time the previously described effects of **1** in HDAC3 and PPARγ signaling, opening a new research field for the therapeutic potential of this compound family.

Psammaplins are a compound family from marine sponges that have attracted much attention due to their bioactivities and unique chemical structures. Psammaplin A (**1**) was the first symmetrical bromotyrosine dimer identified.^1,2^ Because of this singular structure, the pharmacological activity of **1** has been widely studied. The compound has shown antibacterial, antiviral, and cytotoxic effects, among others.^3−5^ Along with **1**, several derivatives have been described, like psammaplins B, K (**2**), or P and bisaprasin (**3**), the biphenyl dimer of **1**. These analogs have been tested in diverse bioassays focused on their cytotoxic activity and presented distinct potencies due to the structural differences.^4,6,7^ However, the neuroprotective potential of this compound family has not been explored.

The cytotoxic activity of psammaplins has been attributed to their ability to inhibit class I histone deacetylases (HDACs).^6,7^ These enzymes regulate transcriptional repression through chromatin condensation and are divided into four classes. Class I HDACs are Zn-dependent enzymes that include HDACs 1, 2, 3, and 8. Their inhibition has been also proposed as a therapeutic strategy for neurodegenerative diseases.^8,9^ HDAC3 is the most abundant isoform in the brain, and its repression has shown promising effects against neurodegeneration.^10,11^ One of the consequences of HDAC3 inhibition is the activation of peroxisome proliferator-activated receptor gamma (PPARγ), which regulates genes involved in lipid metabolism, antioxidant defense, and anti-inflammatory signaling.^11^ PPARγ is a ligand-activated transcription factor that belongs to the nuclear hormone superfamily and upregulates neuroprotective proteins like nuclear factor E2-related factor 2 (Nrf2), superoxide dismutase (SOD), catalase (CAT), or glutathione peroxidase (GPx).^12^ Furthermore, PPARγ is implicated in mitochondrial function, as it regulates the electron transport chain and mitochondrial biogenesis.^13,14^ Compound **1** has been described as an activator of PPARγ, but this effect was related to the antitumor activity of the molecule.^15^

Mitochondrial dysfunction and oxidative stress play a key role in the onset of neurodegenerative illnesses like Parkinson’s and Alzheimer’s diseases. Aging leads to an augmentation in reactive oxygen species (ROS) release and to a reduction in antioxidant systems efficacy that generates an oxidative environment that affects proteins, lipids, and nucleic acids.^16^ ROS accumulation enhances mitochondrial dysfunction through the opening of mitochondrial permeability transition pore (mPTP), which dissipates the mitochondrial membrane potential (ΔΨm) and can produce the collapse of the organelle, finally leading to neuronal death.^17^ As mitochondrial dysfunction and oxidative stress are early events in neurodegeneration, pharmacological approaches directed to improve the intrinsic antioxidant defense of neurons and to enhance mitochondrial function, like PPARγ activation, are promising strategies for counteracting these pathologies.^18,19^

In this sense, the already described effect of **1** on PPARγ, along with the capacity of psammaplins to inhibit class I HDACs, makes these compounds promising candidates for the treatment of neurodegeneration. In this work, **1** and its two derivatives, **2** and **3** (Figure 1), isolated from the marine sponge *Aplysinella rhax*, were tested in an *in vitro* model of oxidative stress in SH-SY5Y human neuroblastoma cells in order to disclose their neuroprotective potential.

**Figure 1:**
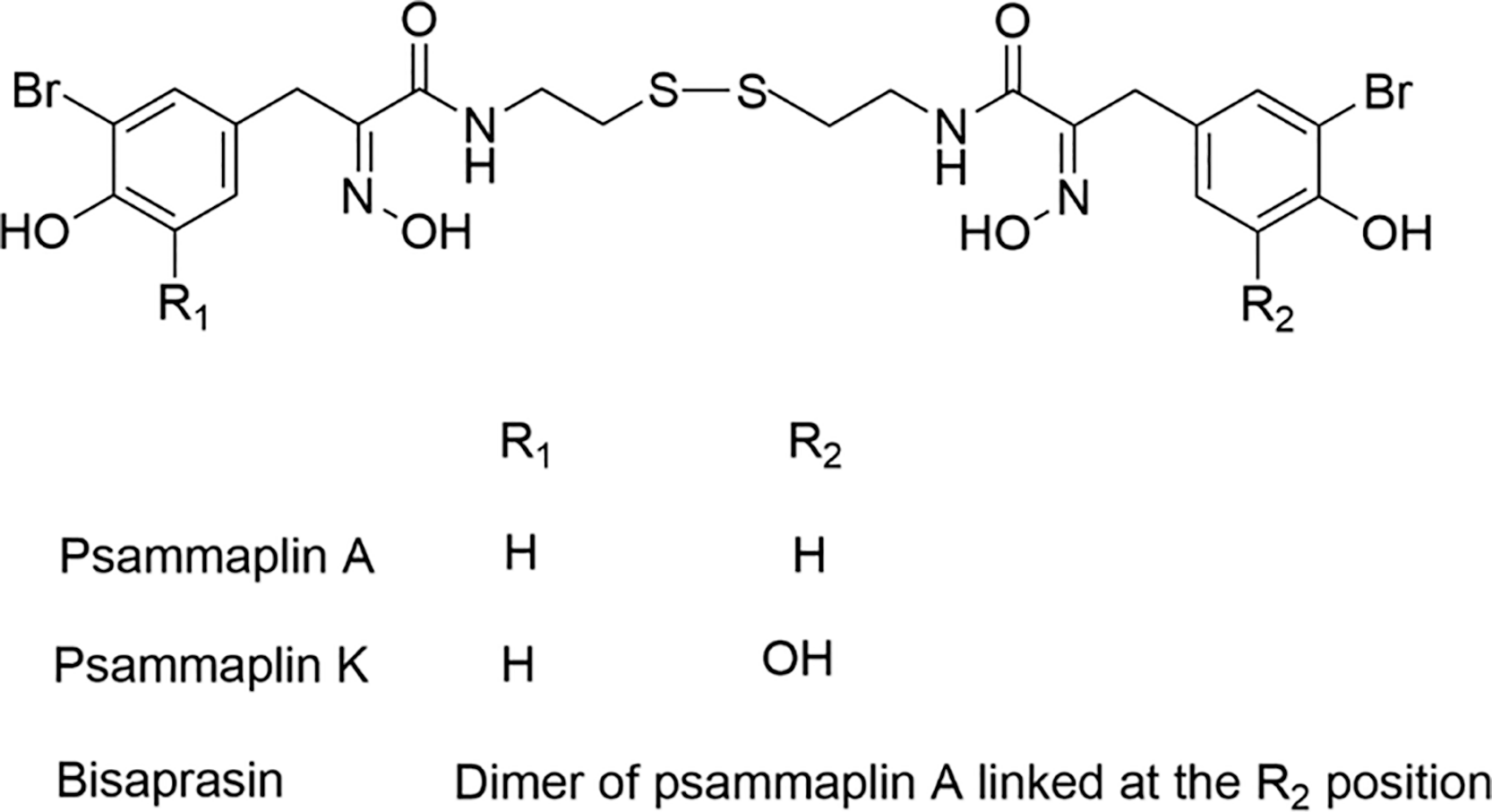
Chemical structures of compounds **1** (psammaplin A), **2** (psammaplin K), and **3** (bisaprasin).

## Results and Discussion

### Effect of Psammaplins on PPARγ Activity

At first, the effects of compounds **1**–**3** on cell viability were tested. SH-SY5Y cells were treated at 0.001, 0.01, 0.1, and 1 μM for 24 h, and an MTT assay was performed. None of the compounds reduced cell viability at these concentrations, so neuroprotective assays were carried out at the same doses (Figure S1).

**Figure 2:**
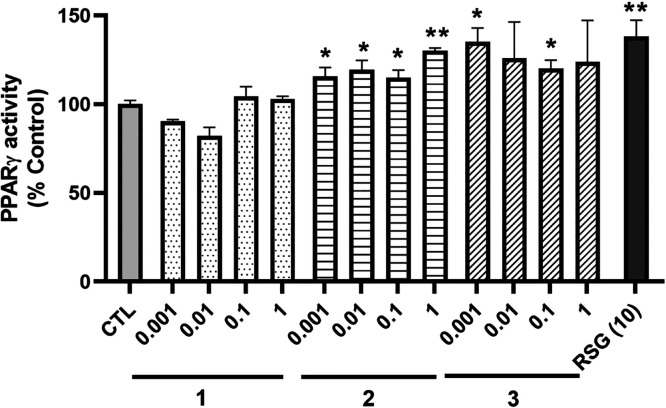
Activity of PPARγ in the nucleus after treatment with *A. rhax* metabolites. SH-SY5Y cells were treated with compounds at nontoxic concentrations for 6 h and lysed, and the activity of PPARγ was evaluated with a commercial kit. Rosiglitazone (RSG) at 10 μM was used as the positive control. Data are mean ± SEM of three independent replicates performed by triplicate. Results are expressed as percentage of control cells and compared by a one-way ANOVA test followed by Dunnett’s post hoc test (*p < 0.05, **p < 0.01 compared to control cells).

In view of the previous results about **1** and PPARγ in a different cell line, the ability of the compounds to activate this transcription factor was analyzed.^15^ With this purpose, cells were lysed after treatment with **1**–**3** for 6 h, and nuclear extracts were used to determine PPARγ activity with a commercial kit. As Figure 2 shows, **2** was able to increase the activity of PPARγ at all the concentrations tested, reaching levels of 130 ± 2% (*p* < 0.01) at 1 μM. Regarding **3**, it also augmented the transcription factor activity at 0.001 and 0.1 μM (*p* < 0.05). As expected, the positive control rosiglitazone (RSG) increased PPARγ activation to 138 ± 9% (*p* < 0.01), compared to control cells.

### Evaluation of the Antioxidant Potential of Compounds **1**–**3**

**Figure 3:**
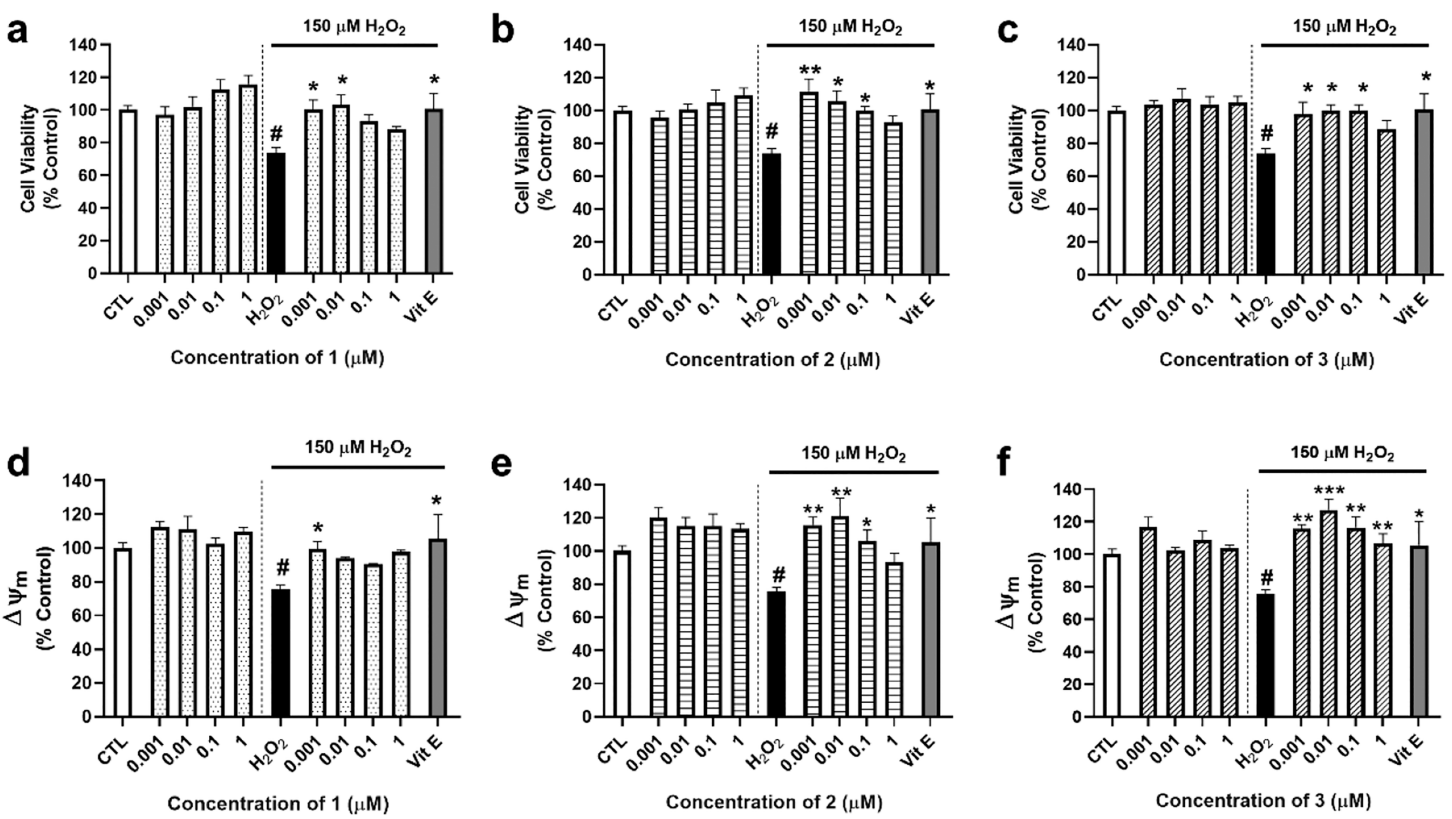
Effects of **1**–**3** on cell viability and mitochondrial membrane potential. Human neuroblastoma cells were treated with compounds with and without 150 μM H_2_O_2_ for 6 h. Their effects on cell viability were assessed with the MTT assay, while ΔΨm was determined by TMRM dye. Cell viability after treatment with (a) **1**, (b) **2**, and (c) **3**. Effects of (d) **1**, (e) **2**, and (f) **3** on ΔΨm. Vitamin E (Vit E) at 25 μM was used as a positive control. Mean ± SEM of three independent replicates was performed by triplicate. Data are expressed as percentage of untreated control cells. Statistical differences were determined by one-way ANOVA and Dunnett’s tests (#*p* < 0.05 compared to control cells; *p < 0.05, **p < 0.01, and ***p < 0.001 compared to H_2_O_2_ control cells).

Due to the role of PPARγ in the regulation of antioxidant enzymes, the experiments were continued by analyzing the protective effect of compounds in an *in vitro* model of oxidative stress. For these assays, SH-SY5Y cells were cotreated with the compounds at concentrations ranging from 0.001 μM to 1 and 150 μM H_2_O_2_ for 6 h.^20^ Then, their effect on cell viability and ΔΨm was analyzed (Figure 3).

Compound **1** protected neuronal cells from the loss of cell viability produced by 150 μM H_2_O_2_ (76 ± 4%, *p* < 0.05 compared to control cells) at 0.001 and 0.01 μM, with levels of 100 ± 6% and 96 ± 5% (*p* < 0.05 compared to H_2_O_2_ control), respectively (Figure 3a). With respect to **2** and **3**, these compounds also presented neuroprotective effects, in this case at 0.001, 0.01, and 0.1 μM (Figure 3b,c). As expected, the antioxidant compound vitamin E (Vit E) at 25 μM, used as a positive control, improved cell viability up to 106 ± 11% (*p* < 0.05). Next, tetramethylrhodamine methyl ester (TMRM) was used to assess ΔΨm. The addition of the oxidant induced a depolarization of mitochondria (76 ± 2%, *p* < 0.05 with respect to control cells) that was reversed by **1** at 0.001 μM (100 ± 4%), **2** at 0.001, 0.01, and 0.1 μM, and **3** at all the concentrations assayed (Figure 3d–f).

**Figure 4:**
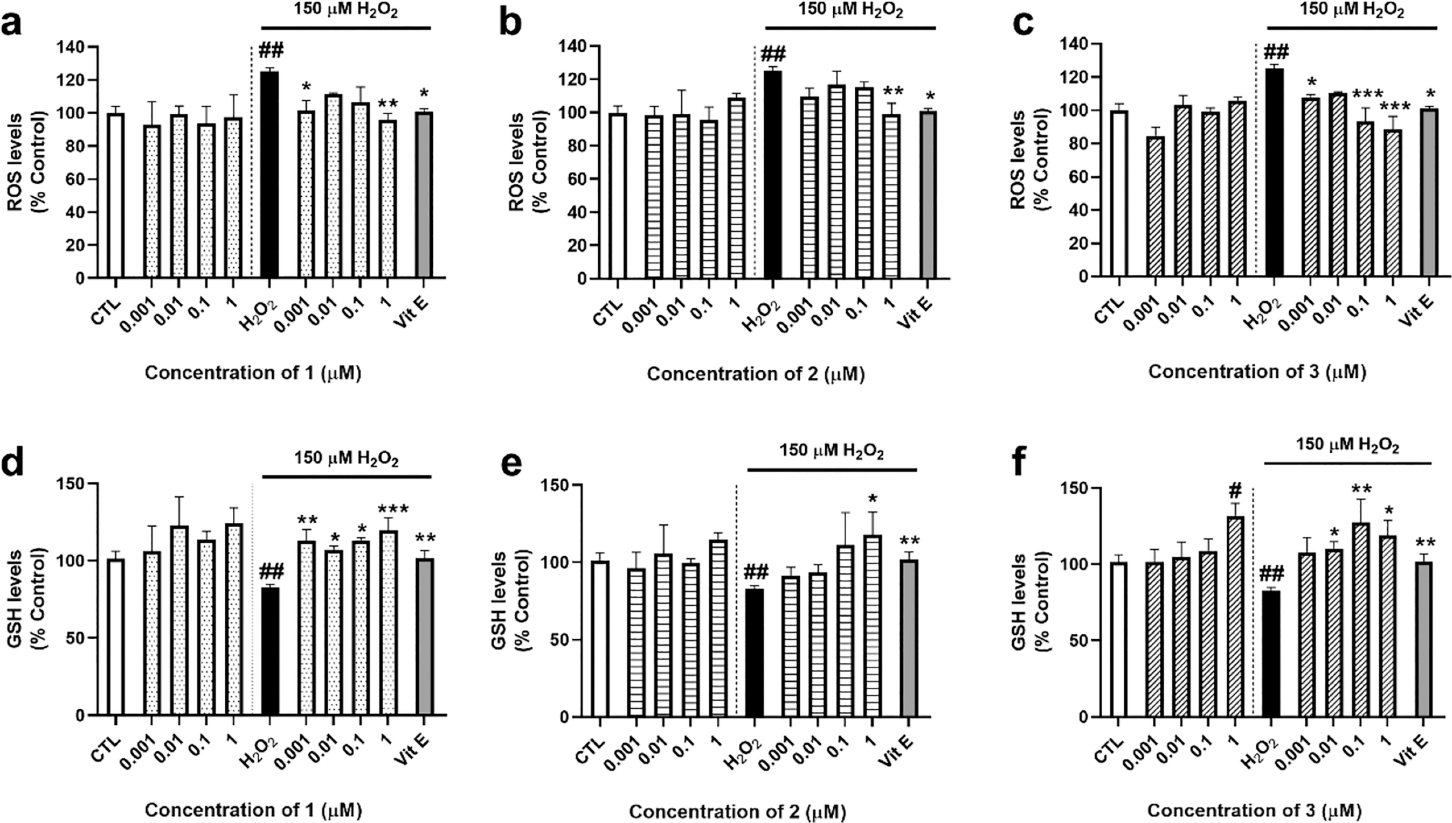
ROS and GSH levels after treatment with compounds. *A*. *rhax* metabolites and 150 μM H_2_O_2_ were added to the SH-SY5Y cells for 6 h. Then, ROS and GSH levels were determined with the fluorescent probes carboxy-H_2_DCFDA and Thiol Tracker Violet, respectively. Effects of (a) **1**, (b) **2**, and (c) **3** on intracellular ROS levels. GSH content after addition of (d) **1**, (e) **2**, and (f) **3**. Vitamin E (Vit E) at 25 μM was used as a positive control. Data presented as mean ± SEM of three replicates carried out in triplicate and expressed as percentage of untreated control cells. Statistical significance was assessed by one-way ANOVA followed by Dunnett’s post hoc test (##*p* < 0.01 compared to control cells; *p < 0.05, **p < 0.01, and ***p < 0.001 compared to H_2_O_2_ control cells).

When ROS levels were determined, it was observed that 150 μM H_2_O_2_ increased the release of these toxic molecules to 125 ± 2% (*p* < 0.01, with respect to control cells). Addition of **1** at 0.001 and 1 μM significantly reduced ROS levels (101 ± 6% and 96 ± 4%, respectively) (Figure 4a). Compound **2** only produced significant effects at 1 μM (99 ± 7%, *p* < 0.01 compared to cells treated with H_2_O_2_ alone) (Figure 4b), while **3** diminished ROS release at 0.001, 0.1, and 1 μM, showing levels between 97% and 87% (Figure 4c), similar to the effect produced by Vit E (101 ± 2%, *p* < 0.05).

As glutathione (GSH) is the main nonenzymatic antioxidant in cells, the study was followed by determining its levels after treatment with *A. rhax* metabolites. Addition of 150 μM H_2_O_2_ reduced GSH content to 83 ± 2% (*p* < 0.01, compared to control cells) (Figure 4d–f). Compound **1** was able to recover the antioxidant levels at all the concentrations tested, reaching a percentage of 120 ± 8% at 1 μM (*p* < 0.001, with respect to H_2_O_2_ control) (Figure 4d). Compound **2** presented significant results at the same concentration (117 ± 15%, *p* < 0.05 compared to H_2_O_2_ control) (Figure 4e). In the case of **3**, it induced an increase in GSH levels when cells were treated with the compound alone at 1 μM (*p* < 0.05, with respect to control). Further, **3** augmented GSH content under oxidative stress conditions when cells were treated at 0.01, 0.1, and 1 μM (Figure 4f).

### Assessment of Mitochondrial Permeability Transition Pore after Treatment with **1**–**3**

**Figure 5:**
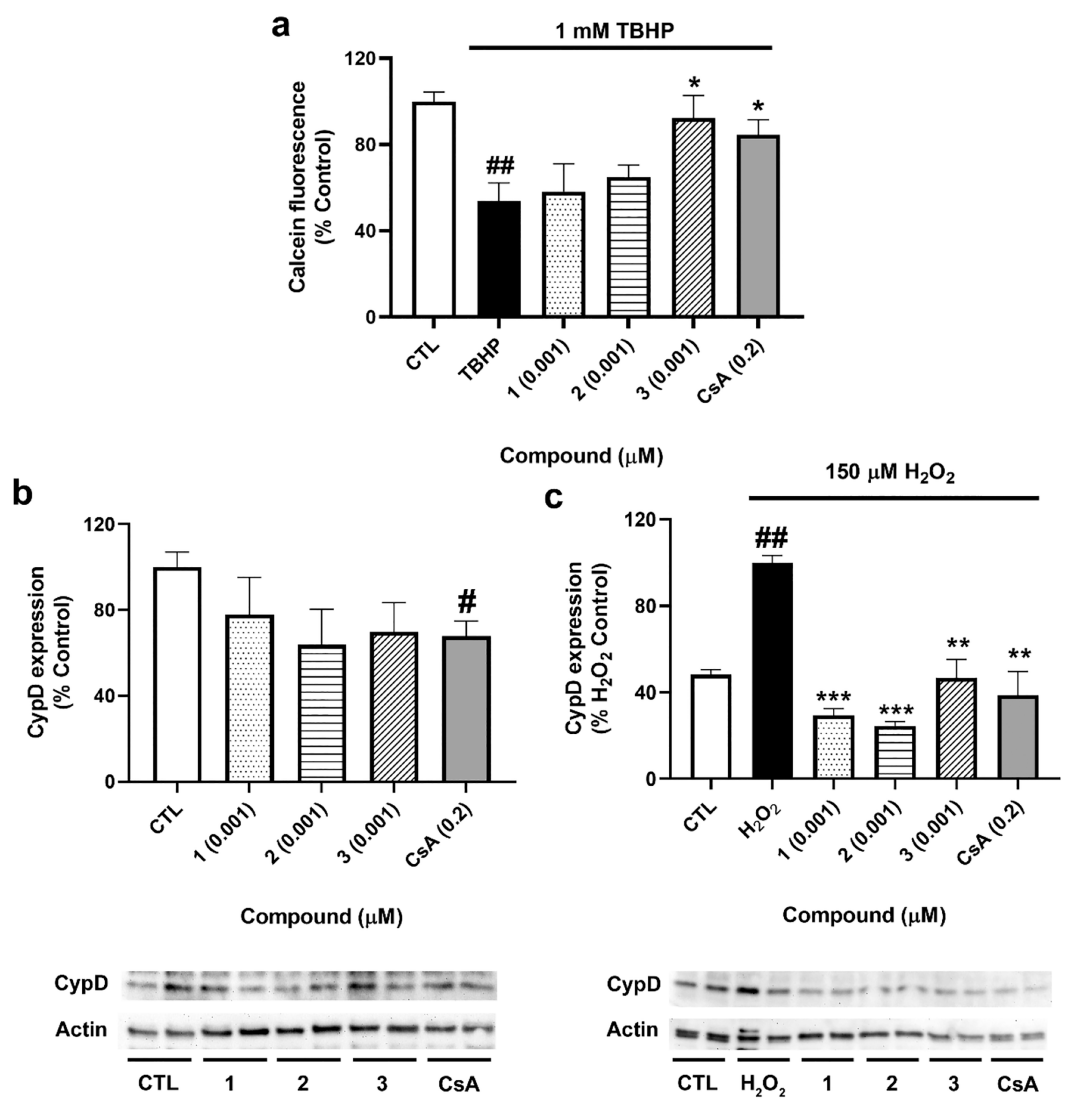
Evaluation of mPTP after treatment with **1**–**3**. (a) Determination of the mPTP opening. SH-SY5Y cells were loaded with calcein-AM and CoCl_2_ and treated with compounds and 1 mM TBHP, and fluorescence was measured by flow cytometry. Cyclosporine A (CsA) (0.2 μM) was used as a positive control. Data are mean ± SEM of three independent experiments and presented as percentage of control cells. Statistical differences determined by one-way ANOVA and Dunnett’s tests (##*p* < 0.01 compared to control cells; *p < 0.05 compared to cells treated only with TBHP). (b) Effect of compounds on CypD expression. (c) Expression of CypD after the addition of *A. rhax* metabolites and 150 μM H_2_O_2_. Cells were treated for 6 h, and the expression of CypD was analyzed by Western blot. Cyclosporine A (CsA) at 0.2 μM was used as a positive control. Protein band expression was normalized by actin levels. Mean ± SEM of three replicates carried out by duplicate and expressed as percentage of untreated control cells and H_2_O_2_ control, respectively. Statistical significance was analyzed by one-way ANOVA and Dunnett’s tests (##*p* < 0.01 compared to control cells; **p < 0.01 and ***p < 0.001 compared to cells treated with H_2_O_2_ alone).

In view of the effects of the compounds on ΔΨm, their capacity to inhibit the opening of mPTP was determined. For this assay, the minimal effective concentration in the oxidative stress model was selected, 0.001 μM. As Figure 5a shows, *tert*-butyl hydroperoxide (TBHP) reduced calcein fluorescence to 54 ± 8% (*p* < 0.01, compared to control cells), so the oxidant induced mPTP opening. Treatment with **3** recovered the signal to 92 ± 11% (*p* < 0.05, compared to cells treated with TBHP), a higher value than that obtained in cells treated with the positive control cyclosporine A (CsA) (85 ± 7%, *p* < 0.05).

These results were further confirmed by analyzing the expression of cyclophilin D (CypD), the main regulator of the mPTP opening.^17^ After treatment with compounds for 6 h with and without 150 μM H_2_O_2_, cells were lysed and the expression of the protein was evaluated by Western blot. When cells were treated with **1**–**3** alone, no significant effects on CypD expression were observed; only the positive control CsA decreased its levels (68 ± 7%, *p* < 0.05) (Figure 5b). However, under oxidative stress conditions, **1**–**3** significantly diminished CypD expression to 30 ± 3 (*p* < 0.001), 25 ± 2 (*p* < 0.001), and 47 ± 9% (*p* < 0.01), respectively (Figure 5c), confirming their effect on mPTP blockade.

### Effects of Compounds on PPARγ Translocation and Its Downstream Signaling

**Figure 6:**
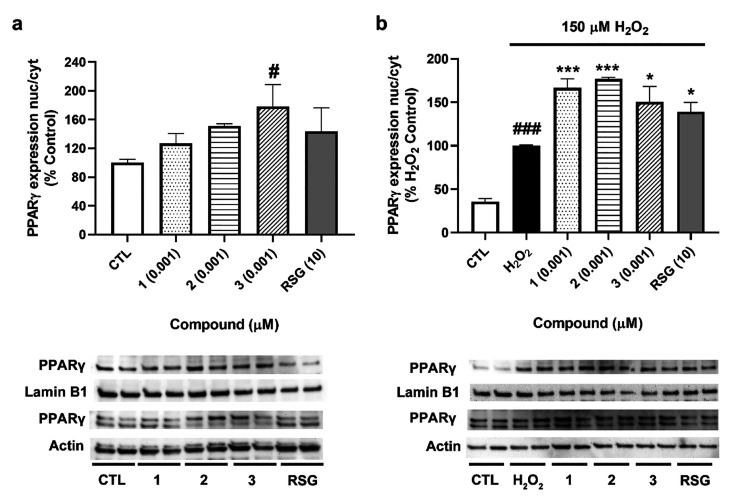
Effects of compounds on PPARγ translocation. Cells were treated with **1**–**3** for 6 h, and the expression of the transcription factor was assessed by Western blot. (a) Expression of PPARγ after treatment with compounds. (b) Effects of *A. rhax* metabolites in PPARγ translocation under oxidative stress conditions. Translocation of PPARγ was determined as the ratio between nuclear and cytosolic levels. Protein band expression was normalized by lamin B1 and actin levels in the nuclear and cytosolic fractions, respectively. Values are mean ± SEM of three replicates carried out by duplicate and presented as percentage of control cells or H_2_O_2_ control. Statistical differences were determined by one-way ANOVA and Dunnett’s tests (#*p* < 0.05, ###*p* < 0.001 compared to control cells; *p < 0.05, ***p < 0.001 compared to cells treated with H_2_O_2_ alone).

Next, to determine if psammaplins were able to induce the translocation of PPARγ to the nucleus, its expression was analyzed in both cytosolic and nuclear fractions (Figure 6).

As can be observed in Figure 6a, compound **3** at 0.001 μM was able to significantly increase PPARγ translocation (180 ± 30%, *p* < 0.05 compared to control cells). Compounds **1**–**2** also increased the transcription factor translocation to 136 ± 10% and 151 ± 3%, respectively, although this augmentation did not reach statistical significance. Under oxidative stress conditions, the three compounds significantly augmented PPARγ translocation to the nucleus, with levels between 150% and 177% of the H_2_O_2_ control. These values were higher than the increase produced by RSG (139 ± 11%, *p* < 0.05) (Figure 6b).

**Figure 7:**
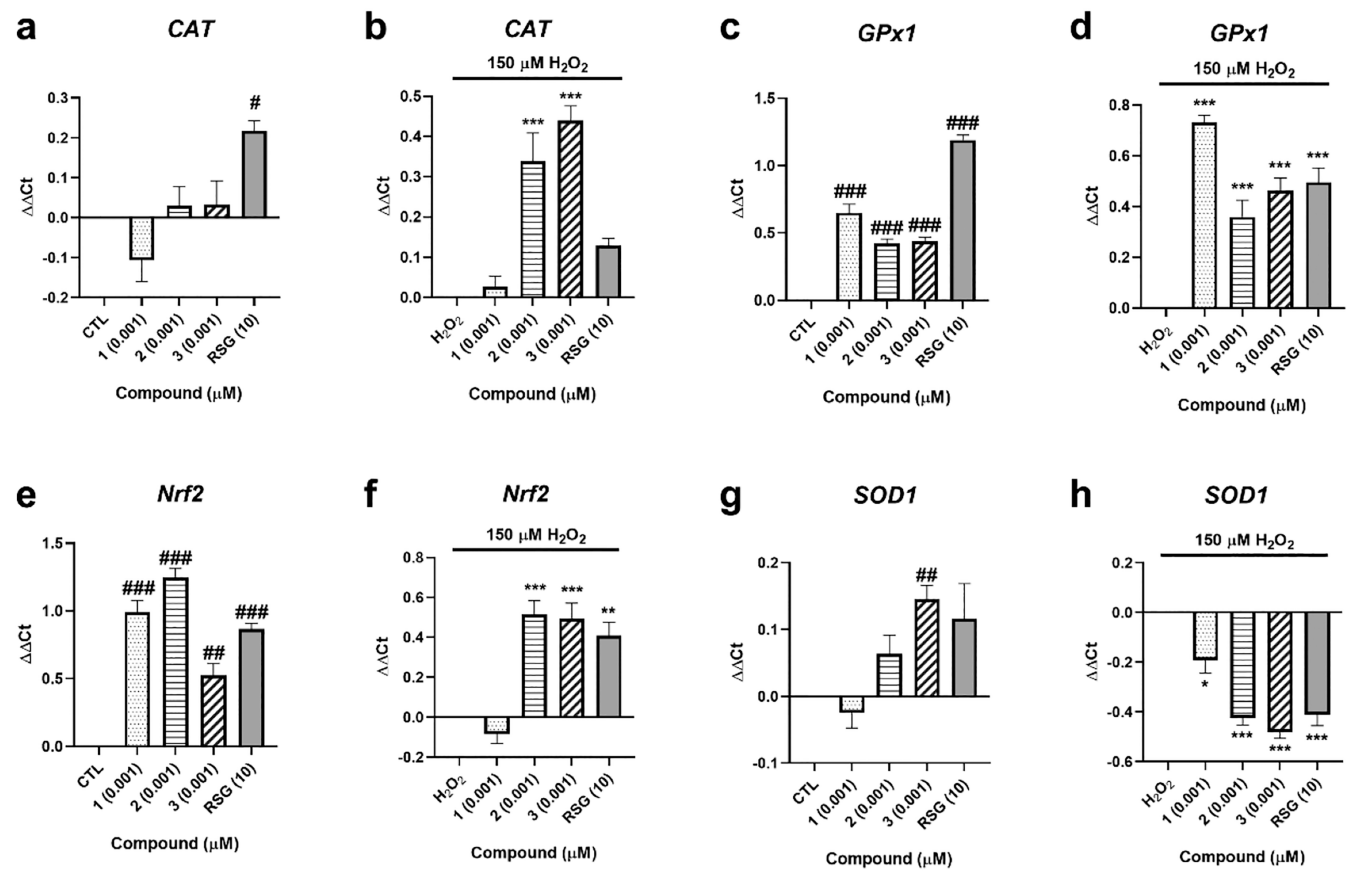
Gene expression of antioxidant enzymes after treatment with *A. rhax* metabolites. Relative gene expression of *CAT* after 6 h of treatment with compounds without (a) and with (b) 150 μM H_2_O_2_, *GPx1* when SH-SY5Y cells were treated with **1**–**3** for 6 h (c) and injured with 150 μM H_2_O_2_ (d), *Nrf2* after addition of compounds (e) and cotreatment with compounds and H_2_O_2_ (f), and *SOD1* when metabolites were added to cells under physiological (g) and oxidative stress (h) conditions. Rosiglitazone (RSG) at 10 μM was used as a positive control. Relative gene expression was calculated with the ΔΔCt method. Control cells and H_2_O_2_ control were used as calibrator, and *RPL0* was the internal normalization control. Data are expressed as the mean ± SEM of three independent replicates performed by triplicate. Statistical significance evaluated by one way ANOVA and Dunnett’s tests (#*p* < 0.05, ##*p* < 0.01, ###*p* < 0.01, compared to control cells; *p < 0.05, **p < 0.01, ***p < 0.001, compared to cells treated with H_2_O_2_ alone).

In view of the ability of compounds to activate PPARγ, the study was continued by determining the expression of genes regulated by this transcription factor and involved in cell antioxidant defense (Figure 7). Regarding *catalase* (*CAT*), when cells were treated with compounds alone, only RSG was able to increase its expression (Figure 7a). Under oxidative injury, both **2** and **3** significantly augmented *CAT* expression (*p* < 0.001) (Figure 7b). *Glutathione peroxidase 1* (*GPx1*) was significantly increased after treatment with **1**–**3** both in the presence and in the absence of 150 μM H_2_O_2_ (Figure 7c,d). With respect to *nuclear factor E2-related factor 2* (*Nrf2*), its expression was augmented after addition of the three compounds at 0.001 μM (Figure 7e), while only **2** and **3** produced significant effects under oxidative stress conditions (Figure 7f). Finally, *superoxide dismutase 1* (*SOD1*) expression was analyzed, finding that only **3** increased its levels when cells were treated only with compounds (Figure 7g). The gene expression of this enzyme was decreased by the three *A. rhax* metabolites, as well as by RSG, after cotreatment with compounds and 150 μM H_2_O_2_ (Figure 7h).

### Analysis of *A. rhax* Metabolites Effects on HDAC3 Activity

**Figure 8:**
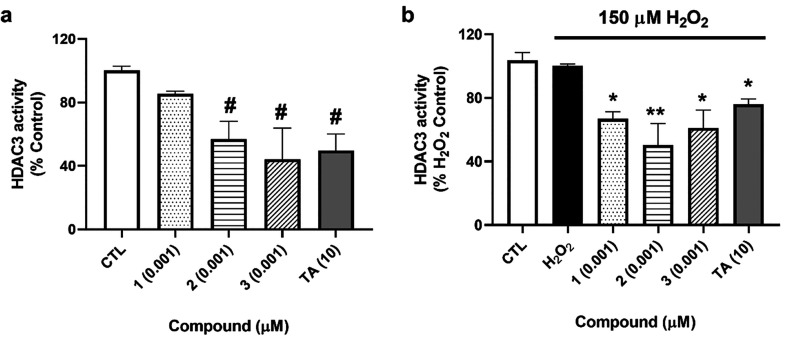
Effects of **1**–**3** on HDAC3 activity. SH-SY5Y cells were treated with the compounds and 150 μM H_2_O_2_ for 6 h and lysed, and the activity of HDAC3 was determined in nuclear fractions with a commercial kit. (a) Activity of HDAC3 after treatment with compounds alone. (b) Nuclear activity of HDAC3 after cotreatment with *A. rhax* metabolites and H_2_O_2_. Trichostatin A (TA) at 10 μM was used as a positive control. Mean ± SEM of three replicates carried out by duplicate. Values expressed as percentage of control and H_2_O_2_ control cells, respectively. One-way ANOVA and Dunnett’s tests were used for analyzing statistical differences (#*p* < 0.05, compared to control cells; *p < 0.05, **p < 0.01, compared to cells treated with H_2_O_2_ alone).

Finally, due to the role of HDAC3 in PPARγ repression and the previously described activity of psammaplins as class I HDAC inhibitors, the effects of compounds on HDAC3 activity were assessed.^6,11^ As it is believed that HDAC3 suppresses PPARγ gene expression when they are in the nucleus, SH-SY5Y cells were lysed and nuclear fractions were used to determine HDAC3 activity with a commercial kit.^21^ Compounds **2** and **3** decreased HDAC3 activity to 57 ± 11% and 44 ± 19% (*p* < 0.05), respectively (Figure 8a), when cells were treated with compounds at 0.001 μM. When cells were damaged with H_2_O_2_, the three compounds were able to inhibit HDAC3 activity to levels between 50% and 67%, a greater decrease than the effect obtained with trichostatin A (TA) at 10 μM (77 ± 4%, *p* < 0.05) (Figure 8b).

The incidence of neurodegenerative diseases is dramatically increasing worldwide. The most common dementia, Alzheimer’s disease, has doubled its mortality in the past few years in Europe, and the number of cases is predicted to reach 18.65 million in 2050.^22^ Current therapies are symptomatic treatments, and no disease-modifying drugs are available; therefore, there is a need for new pharmacological strategies. The look for new targets that improve mitochondrial function and decrease inflammation and oxidative stress has attracted much attention, as these processes are altered at initial stages of the illnesses.^23^ The crucial role of PPARγ in the regulation of antioxidant defense and the modulation of oxidative phosphorylation and mitochondrial biogenesis make the activation of the transcription factor a promising strategy for neurodegeneration.^12^

In this work, we describe for the first time the neuroprotective activity of psammaplin A (**1**) and its analogs psammaplin K (**2**) and bisaprasin (**3**). These compounds diminished the cell death induced by oxidative damage, recovering ΔΨm and GSH levels and decreasing ROS content, an effect mediated by PPARγ activation. Regarding **2** and **3**, they induced an increase on PPARγ activity; however, when its nuclear and cytosolic expression was determined, only **3** produced a significant augmentation on PPARγ translocation. The three compounds induced an important augmentation in PPARγ translocation under oxidative stress conditions, which suggests a higher efficiency under pathological circumstances. PPARγ is a ligand-activated transcription factor whose activity is not only regulated by ligands but also posttranslational modifications such as phosphorylation and acetylation are implicated in its activation.^11,24^ Therefore, the differences found among PPARγ activity and expression after treatment with **2** seem to be related to an increase in the transcription factor activity without affecting its nuclear expression. In the case of compound **1**, it did not affect PPARγ activity and expression without the presence of H_2_O_2_. However, compound **1** activated transcription factor translocation to the nucleus when the oxidant was added, agreeing with the higher efficiency found with **2** and **3** treatments.

As a consequence of PPARγ activation, we observed that psammaplins augmented *Nrf2*, *CAT*, and *GPx1* expression. When Nrf2 is activated, it binds to the antioxidant response elements and induces the expression of antioxidant enzymes, improving the effect produced by PPARγ activation.^25^ In these experiments, the compounds also presented better effects when oxidative stress was induced. Together with their involvement in the regulation of antioxidant genes, PPARγ and Nrf2 activation is also related to mitochondrial function. Both transcription factors modulate the electronic transport chain and the subsequent maintenance of ΔΨm.^26,27^ Thus, the observed effect of the compounds on the reduction of CypD expression under oxidative stress conditions seems to be due to a decrease in mPTP opening when psammaplins were present. However, only **3** was able to block the pore when it was analyzed by flow cytometry. These differences could be related to the distinct incubation times among both assays, as CypD expression was determined after an incubation of 6 h, and the mPTP opening was evaluated after 10 min of treatment. In fact, when ΔΨm was assessed, the three compounds repolarized the mitochondria at 1 nM after 6 h of incubation, agreeing with the results obtained in CypD expression.

In the absence of ligands, PPARγ binds to the nuclear corepressor formed by HDAC3 and the silencing mediator for retinoic and thyroid hormone/nuclear receptor corepressor. Therefore, the inhibition of HDAC3 leads to the acetylation and activation of the transcription factor and the consequent increase on antioxidant enzyme expression.^11^ Because psammaplins have been widely described as class I HDAC inhibitors, their effect on HDAC3 activity in SH-SY5Y cells was analyzed, finding that **2** and **3** decreased the enzyme activity under physiological conditions, agreeing with the effects observed on PPARγ. Again, compounds were more active after oxidative injury as the three psammaplins inhibited the enzyme when H_2_O_2_ was added. Therefore, it seems that the effect on PPARγ is due to the ability of psammaplins to inhibit HDAC3, an activity that is enhanced under oxidative stress conditions. The enzyme is expressed in nucleus and cytosol, and oxidative damage promotes its translocation to the nucleus and strengthens its association to PPARγ, which could explain the greater effect of compounds when H_2_O_2_ was present.^28^ HDAC3 repression has shown promising results in cellular and animal models of neurodegeneration; however, most HDAC3 inhibitors usually also target other HDAC isoforms, producing side effects.^29^ In this sense, **1** has shown selectivity toward class I HDACs, whereas **2** and **3** effects on other isoforms remain unknown.^2,7,30,31^ Future studies should disclose the selectivity of analogs, as well as the effect of **1** on HDAC1, -2, and -4 in neuronal cells, in order to better understand their potential as neuroprotective drugs.

Psammaplins have been widely described as pro-apoptotic compounds, an activity that occurs at concentrations in the high micromolar range.^1,5,32^ Recently, it has been reported that 10 μM PsA inhibits the development of bovine embryos through the induction of oxidative stress.^33^ In our study, *A. rhax* metabolites were tested at lower and nontoxic concentrations, finding that these doses were enough to induce a neuroprotective effect. Other natural compounds, especially polyphenols, have also presented this biphasic behavior, showing protective and cytotoxic outcomes depending on the concentration.^34,35^ Moreover, we have previously observed a similar effect for another sponge-derived molecule, jasplakinolide, known for its pro-apoptotic properties.^36^

Compound **3**, the biphenyl dimer of **1**, was the most effective compound in all of the assays. It has been proposed that **1** acts as a prodrug; when it enters the cells and is reduced, the disulfide bridge is broken, and two thiol groups are formed.^6^ These reactive groups have been proposed as being responsible for class I HDAC inhibition, since the enzymes have a Zn in their catalytic pocket. Monomers of **1** have been synthesized, finding that they retain the activity of **1** but with lower half inhibitory concentration on HDAC activity assays.^2^ As compound **3** has two disulfide bonds that can be reduced inside the cells, it would produce four thiol residues that could be responsible for the higher activity of the compound. Due to the need of psammaplins reduction inside the cells, the amount of GSH and thioredoxin has been shown to be critical to their activity. Compound **1** activity was decreased in GSH-depleted cells, and the reduction of the compound leads to higher activity in HDAC activity assays.^6,31^ Moreover, when **1** is oxidized before cell treatment, its activity is abolished.^6^ In our model, an oxidative environment was induced with H_2_O_2_, which reduced GSH levels 17%. This decrease does not seem to affect psammaplins activity; on the contrary, compounds had greater effects when oxidative stress was generated. The mentioned studies used an inhibitor of gamma-glutamylcysteine synthetase that produced a great depletion of GSH levels. Further, addition of 150 μM H_2_O_2_ to the cells does not reduce *A. rhax* metabolites activity. In the previous study, H_2_O_2_ at a high concentration (1%) was used to oxidize **1** before performing the assays.^6^ In view of our results, it seems that a small decrease in GSH cell levels and the addition of H_2_O_2_ at low concentrations do not alter the capacity of cells to reduce the compounds to the active monomers.

In conclusion, **1**–**3** display neuroprotective effects against oxidative stress mediated by their capacity to inhibit HDAC3 and to activate PPARγ and the endogenous antioxidant defense of cells. This new activity of psammaplins makes them candidates for the treatment of illnesses in which these enzymes have been proposed as promising targets, including not only neurodegenerative diseases but also metabolic or cardiovascular pathologies.^26,37^ Therefore, this study opens a novel field of research for this compound family.

## Experimental Section

### Chemicals and Solutions

Tetramethylrhodamine methyl ester (TMRM), Thiol Tracker Violet, 5-(and-6)-carboxy-2′,7′-dichlorodihydrofluorescein diacetate (carboxy-H_2_DCFDA), MitoProbe Transition Pore Assay Kit, Pierce Protease Inhibitor Mini Tablets, Pierce Phosphatase Inhibitor Mini Tablets, phosphate buffered saline (PBS) (pH 7.2), Supersignal West Pico Luminiscent Substrate, Supersignal West Femto Maximum Sensitivity Substrate, oligo-dT primers, RevertAid Reverse Transcriptase, and PowerUp SYBR Green Master Mix were purchased from Thermo Fisher Scientific. RSG, CsA, Nuclear Extraction Kit, PPARγ Transcription Factor Assay Kit, and anti-cyclophilin D (ref ab110324, lot GR3373678-3) and anti-lamin B1 (ref ab16048, lot GR3244890-1) antibodies were obtained from Abcam. Anti-PPARγ (ref MAB3827, lot 2470389) and anti-β-actin (ref MAB1501, lot 3800739) antibodies, HDAC3 Activity Assay Kit, PVDF membrane, and other reagent grade chemicals were purchased from Merck. Locke’s buffer was composed of 154 mM NaCl, 5.6 mM KCl, 1.3 mM CaCl_2_, 1 mM MgCl_2_, 5.6 mM glucose, and 10 mM HEPES. Compounds were dissolved in DMSO, and serial dilutions were done in cell medium. Vehicle concentration was always kept under 0.5% in cell treatments. Control cells were treated with the higher DMSO concentration used in each assay to test the vehicle effect.

### Extraction and Isolation of Compounds

Compounds **1**–**3** were isolated from a marine sponge collected from the Fiji Islands and previously identified as *Aplysinella rhax*. The sponge was collected from the Fiji Islands in December 1997, freeze-dried, and stored at 4 °C. It was identified by Dr. John Hooper of the Queensland Centre for Biodiversity, Queensland Museum, Australia.^38^ A voucher specimen (Voucher number: 9712SD130) is held at the Pacific Regional Herbarium at the University of the South Pacific, Suva, Fiji Islands. Purification was performed using a Waters XSelect C18-CSH 250 × 10 mm HPLC column (Waters Corporation) on an Agilent Technologies 1220 Infinity II HPLC system with a photodiode array detector and using an isocratic solvent system with 80% MeOH/H_2_O (+0.05% TFA) at a flow rate 1.5 mL/min. Compound purity was checked using an Ultrashield Bruker Avance AV400 MHz NMR instrument using CD_3_OD as solvent. NMR data was processed using Mestrenova version 14.3.1 (Mestrelab, Santiago de Compostela, Spain) and compared to data previously described.^3,4,38^ The three compounds showed greater than 96% purity based on relative peak integrations of compound ^1^H NMR signals to contaminant peaks (Figures S2–S4).

### Cell Culture

Human neuroblastoma SH-SY5Y cells were purchased from American Type Culture Collection (ATCC), number CRL2266. Cells were used between passages 10 and 20 and cultured in Dulbecco’s Modified Eagle’s medium: Nutrient Mix F-12 (DMEM/F-12) supplemented with 10% fetal bovine serum, 1% glutamax, 100 U/mL penicillin, and 100 μg/mL streptomycin. Cells were maintained at 37 °C in a humidified atmosphere of 5% CO_2_ and 95% air and dissociated weekly using 0.05% trypsin/EDTA. All the reagents were purchased from Thermo Fisher Scientific. Assays were performed only in undifferentiated SH-SY5Y cells, since this cell line has been widely recognized as a valuable model for oxidative stress. Particularly, undifferentiated cells are more sensitive to oxidative damage and neurotoxins than differentiated neurons, which allows to disclose the neuroprotective potential of compounds against this pathological mechanism.^39−42^

### Cell Viability Assay

The effect of compounds on cell viability was determined by an MTT [3-(4,5-dimethylthiazol-2-yl)-2,5-diphenyltetrazolium bromide] test, as previously described.^36^ SH-SY5Y cells were seeded in 96-well plates at a density of 5 × 10^4^ cells per well. After 24 h, cells were treated with compounds at 0.001, 0.01, 0.1, and 1 μM during 24 h. Next, cells were washed twice with Locke’s buffer, and 500 μg/mL MTT was added to each well. Then, the plate was incubated for 1 h at 37 °C and 300 rpm. After this time, 5% sodium dodecyl sulfate was added to solubilize cells. Finally, the absorbance of formazan crystals was measured at 595 nm in a microplate reader. Quillaja saponin (Merck) at 1 mg/mL was used as cell death control, and its absorbance value was subtracted from the other data.

The MTT assay was also used to determine the neuroprotective abilities of the compounds. With this purpose, cells were seeded as described above and treated with the metabolites at nontoxic concentrations and 150 μM H_2_O_2_ for 6 h. For this experiment, Vit E at 25 μM was used as a positive control.

All of the assays were performed in triplicate three independent times.

### Determination of PPARγ Activity

For this assay, SH-SY5Y cells were seeded at 1 × 10^6^ cells per well in 12-well plates. After 24 h, cells were treated with compounds at concentrations ranging from 0.001 and 1 μM for 6 h. RSG at 10 μM was used as positive control.^19,43^ After incubation, nuclear protein was obtained with a Nuclear Extraction Kit, following the manufacturer’s instructions. Briefly, cells were washed with ice-cold PBS, and a complete hypotonic buffer containing protease and phosphatase inhibitors was added. Cells were incubated for 15 min on ice, and 10% NP-40 was added to each well. Then, samples were centrifuged at 16 100*g* for 1 min at 4 °C, and the supernatant was collected as a cytosolic fraction. The pellet was resuspended in ice-cold complete nuclear extraction buffer supplemented with protease and phosphatase inhibitors. Samples were incubated on ice for 30 min and vortexed in intervals of 15 min. Finally, cell lysates were centrifuged at 16 100*g* for 10 min at 4 °C, and the supernatant was kept as the nuclear fraction. Protein concentration was quantified by the Bradford method.

Then, nuclear fractions were used to determine the effects of compounds on PPARγ activity with the PPARγ Transcription Factor Assay Kit, following the manufacturer’s instructions. The kit is a sensitive ELISA instrument that allows the detection of PPARγ DNA-binding activity. Experiments were carried out three independent times in duplicate, and absorbance values were corrected by protein concentration.

### Analysis of Mitochondrial Membrane Potential

SH-SY5Y cells were seeded at 5 × 10^4^ cells per well in 96-well plates. After 24 h, cells were treated with compounds at nontoxic concentrations and 150 μM H_2_O_2_ for 6 h. Then, cells were rinsed twice with Locke’s buffer, and 1 μM TMRM was added for 30 min at 37 °C and 300 rpm. After this time, cells were lysed with H_2_O and DMSO at 50% and fluorescence was red at 535 nm excitation and 590 nm emission in a microplate reader. Vit E at 25 μM was used as positive control. Assays were performed by triplicate three independent times.^20^

### Measurement of Reactive Oxygen Species and Glutathione Levels

For these assays, SH-SY5Y cells were seeded as described before and allowed to grow for 24 h. Then, cells were treated with compounds at nontoxic concentrations and 150 μM H_2_O_2_ for 6 h.

ROS levels were assessed with carboxy-H_2_DCFDA [5-(and-6)-carboxy-2′,7′-dichlorodihydrofluorescein diacetate]. After treatment, cells were washed twice with a serum-free medium and loaded with 20 μM carboxy-H_2_DCFDA dissolved in a serum-free medium. Cells were incubated for 1 h at 37 °C and 300 rpm, and 200 μL of PBS was added to each well for 30 min at 37 °C and 300 rpm. Next, fluorescence was read at 527 nm excitation, with an emission wavelength of 495 nm.

GSH levels were evaluated with Thiol Tracker Violet, following manufacturer’s instructions. After incubation with compounds and H_2_O_2_, SH-SY5Y cells were rinsed twice with PBS and the dye (10 μM) was added. Then, the plate was incubated at 37 °C and 300 rpm for 30 min, and the fluorescence was read at 404 nm excitation and 526 nm emission in a microplate reader.^20^

All the experiments were carried out in triplicate three independent times, and Vit E at 25 μM was used as positive control.

### Mitochondrial Permeability Transition Pore Assay

The ability of the compounds to block mPTP was evaluated with a MitoProbe Transition Pore Assay Kit, as previously described.^20^ SH-SY5Y cells were seeded in 12-well plates at 5 × 10^5^ cells per well and allowed to grow for 24 h. Then, cells were detached with Detachin solution (Genlatis), washed with PBS, and resuspended in PBS buffer with 0.6 mM CaCl_2_. Cells were loaded with Calcein-AM for 15 min at 37 °C. Next, 0.4 mM CoCl_2_ and compounds at 0.001 μM were added for 15 min at 37 °C. Next, cells were centrifuged, resuspended in calcium-free PBS, and kept on ice. Finally, TBHP at 1 mM was added for 3 min to the cells to induce the mPTP opening. Fluorescence was measured by flow cytometry at 488 nm excitation and 517 nm emission wavelengths with an ImageStream MKII instrument (Amnis Corporation, Luminex Corp). The fluorescence of 10 000 events was analyzed with IDEAS Application vs 6.0 (Amnis Corporation, Luminex Corp). Experiments were performed three independent times, and CsA at 0.2 μM was used as positive control.

### Western Blotting

SH-SY5Y cells were seeded at 1 × 10^6^ cells per well in 12-well plates and allowed to grow for 24 h. After this time, neuroblastoma cells were cotreated with compounds at 0.001 and 150 μM H_2_O_2_ for 6 h. CsA at 0.2 μM and RSG at 10 μM were used as positive controls. Then, cells were washed twice with ice-cold PBS, and 100 μL of a hypotonic buffer was added (20 mM Tris-HCl, pH 7.4, 10 mM NaCl, and 3 mM MgCl_2_, supplemented with phosphatase and protease inhibitors cocktails). Next, cells were incubated on ice for 15 min and centrifuged at 800*g* and 4 °C for 15 min. The supernatant was kept as the cytosolic fraction, and protein concentration was quantified with Direct Detect instrument (Merck). The pellet was dissolved in a nuclear extraction buffer (100 mM Tris at pH 7.4, 2 mM Na_3_VO_4_, 100 mM NaCl, 1% Triton X-100, 1 mM EDTA, 10% glycerol, 1 mM EGTA, 0.1% SDS, 1 mM NaF, 0.5% deoxycholate, and 20 mM Na_4_P_2_O_7_, containing 1 mM PMSF and a protease inhibitor cocktail). Samples were incubated on ice for 30 min, vortexed in intervals of 10 min, and centrifuged at 16 100*g* and 4 °C for 30 min. The supernatant was collected as the nuclear fraction and quantified by the Bradford method.^36^

Electrophoresis was resolved in 4–20% sodium dodecyl sulfate polyacrylamide gels (Biorad), containing 15 μg of cytosolic protein or 10 μg of nuclear protein from each sample. Proteins were transferred to PVDF membranes with Trans-Blot semidry transfer cell (Biorad). Snap i.d. system (Merck) was used for membrane blocking and antibody incubation. CypD was detected with anticyclophilin F primary antibody (1:1000); PPARγ was quantified with anti-PPARγ (1:1000), and Nrf2 was detected with anti-Nrf2 primary antibody (1:1000). Protein band intensity was corrected using anti-β-actin (1:10 000) and anti-lamin B1 (1:5000) in cytosolic and nuclear fractions, respectively. Immunoreactive bands were detected with the Supersignal West Pico Luminiscent Substrate and Supersignal West Femto Maximum Sensitivity Substrate. Diversity GeneSnap system and software (Syngene) were used for protein bands detection. Experiments were performed at least three independent times by duplicate.

### Evaluation of Histone Deacetylase 3 Activity

SH-SY5Y cells were seeded in 12-well plates at 1 × 10^6^ cells per well and treated with compounds at 0.001 and 150 μM H_2_O_2_ for 6 h. After this incubation, cells were lysed as described above for the Western blotting assay. Nuclear fractions were used for the determination of HDAC3 activity with the HDAC3 Activity Assay Kit, following the manufacturer’s instructions. TA at 10 μM was used as positive control, and values were normalized by protein concentration. Experiments were performed three independent times by duplicate.

### Quantitative PCR

SH-SY5Y cells were seeded in 12-well plates at 1 × 10^6^ cells per well and allowed to attach for 24 h. Then, cells were treated with compounds at 0.001 and 150 μM H_2_O_2_ for 6 h. Total RNA was obtained with the HighPurity Total RNA Purification Kit (Canvax Biotech), following the manufacturer’s instructions. RNA purity and concentration were determined with a Nanodrop 2000 spectrophotometer (Thermo Fisher Scientific). cDNA was synthesized with 0.5 μg of RNA, oligo-dT primers, and RevertAid Reverse Transcriptase, following the manufacturer’s instructions. Quantitative PCR was performed using PowerUp SYBR Green Master Mix in a Step-One real-time PCR system (Applied Biosystems). cDNA was amplified with specific primers for *CAT*, *SOD1*, *GPx1*, and *Nrf2* (Table 1). Data were analyzed with the Step-One software (Applied Biosystems). *Ribosomal protein lateral stalk subunit P0* (*RPLP0*) was used as normalization control.^44^ Relative quantification was carried out using the ΔΔCt method using control cells or H_2_O_2_ control as calibrator. All experiments were carried out three independent times in triplicate.

**Table 1:** Primer Sequences Used in qPCR

gene	primer sequence
*Catalase (CAT)*	5′-GAAGTGCGGAGATTCAACACT-3′
	5′-ACACGGATGAACGCTAAGCT-3′
*Glutathione peroxidase 1 (GPx1)*	5′-CCGACCCCAAGCTCATCA-3′
	5′-TTCTCAAAGTTCCAGGCAACATC-3′
*Nuclear factor E2-related factor 2 (Nrf2)*	5′-ACACGGTCCACAGCTCATC-3′
	5′-TGTCAATCAAATCCATGTCCTG-3′
*Superoxide dismutase 1 (SOD1)*	5′-TCATCAATTTCGAGCAGAAGG-3′
	5′-TGCTTTTTCATGGACCACC-3′
*Ribosomal protein lateral stalk subunit P0 (RPLP0)*	5′-GGAGCCAGCGAAGCCACACT-3′
	5′-CACATTGCGGACACCCTCTA-3′

### Statistical Analysis

Data are presented as mean ± SEM. Statistical differences were determined by one-way ANOVA and Dunnett’s post hoc test with Graph Pad Prism 8.0 software. Data were excluded from analysis only when compounds used as a positive control did not work properly. Statistical significance was considered at *p < 0.05, **p < 0.01, and ***p < 0.001.

## Supporting Information Available

^1^H NMR spectra for all compounds and effects of **1**–**3** on cell viability (PDF)
