# Supplementary material for: Psammaplin A and Its Analogs Attenuate Oxidative Stress in Neuronal Cells through Peroxisome Proliferator-Activated Receptor γ Activation
Source: J Nat Prod. 2024 Apr 18;87(4):1187–96. doi: 10.1021/acs.jnatprod.4c00153 (PMC11061836; doi:10.1021/acs.jnatprod.4c00153)
Supplement: Supplementary file 1 — np4c00153_si_001.pdf [file np4c00153_si_001.pdf]

Supporting Information

Psammaplin A and its analogs attenuate oxidative stress in neuronal cells through PPARγ activation

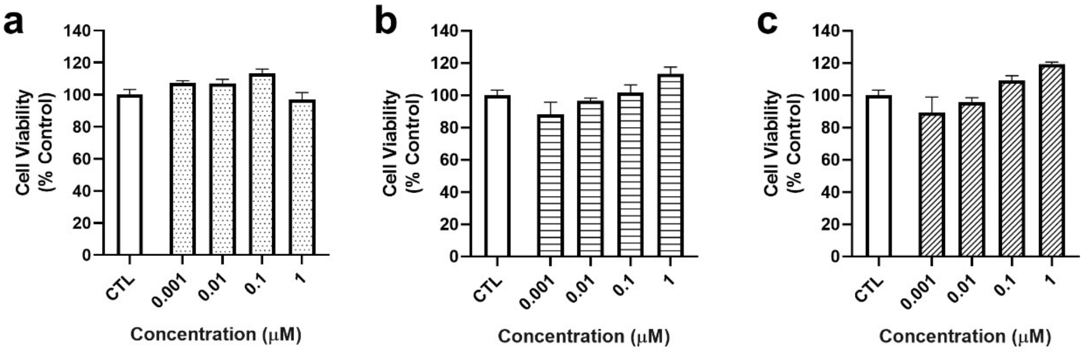

Figure S1. Effect of *A.rhax* metabolites on cell viability. SH-SY5Y cells were treated with compounds for 24 h and their cytotoxic effects were determined by MTT test. (a) **1**, (b) **2** and (c) **3**. Mean±SEM of three replicates carried out by duplicate. Results expressed as percentage of control cells.

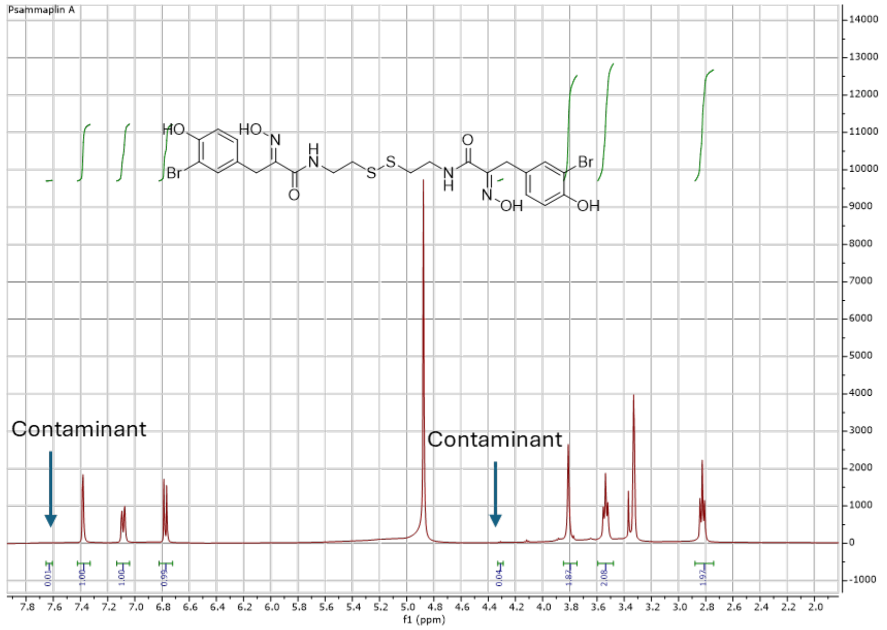

Figure S2. ^{1}H NMR spectrum for **1** in CD_3OD at 400 MHz. Integration of contaminant peaks indicate compound purity is greater than 98%.

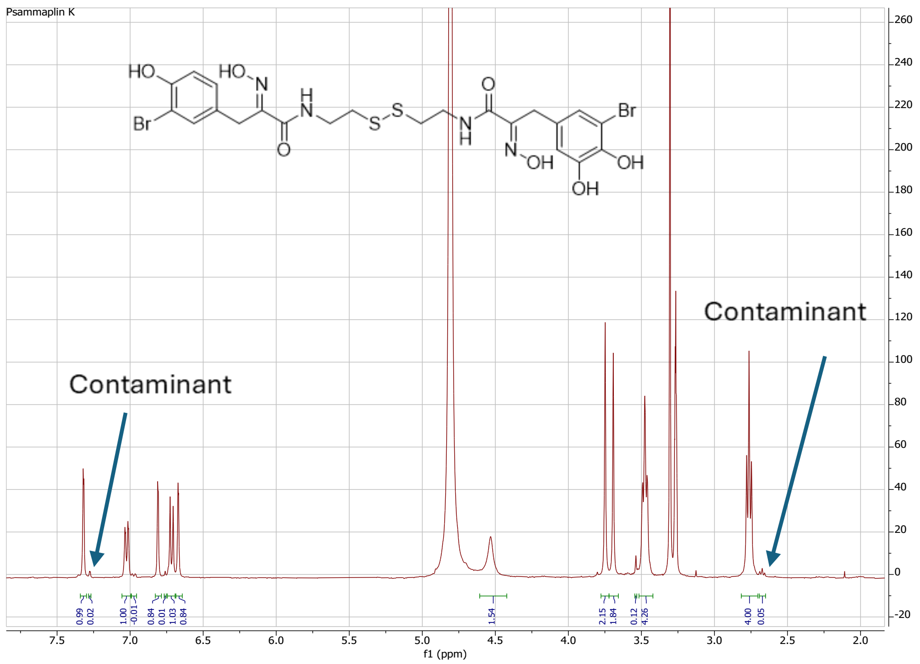

Figure S3. ^{1}H NMR spectrum for **2** in CD_3OD at 400 MHz. Integrations of contaminant peaks indicate compound purity is greater than 96%.

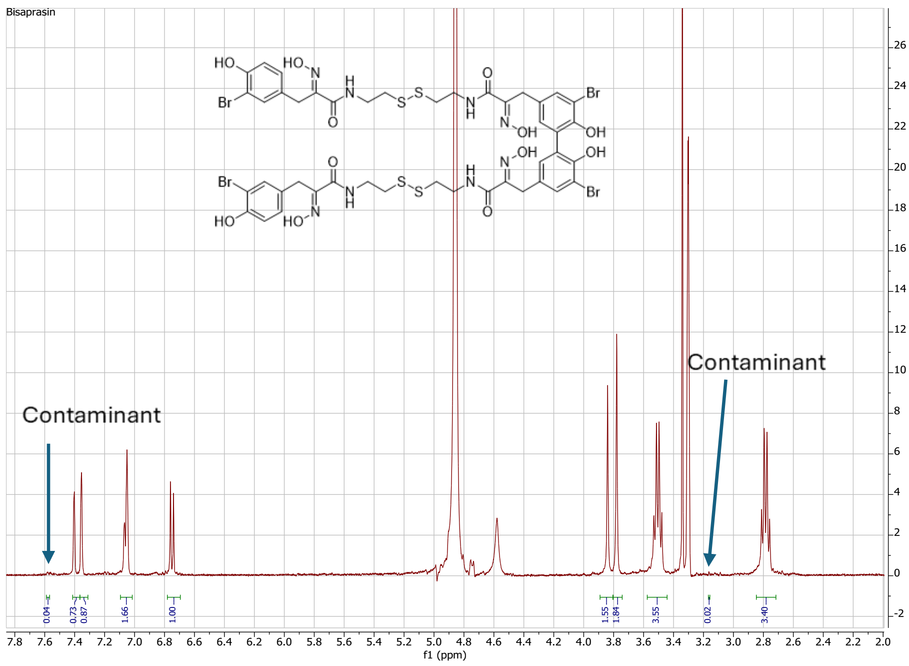

Figure S4. ^{1}H NMR spectrum for **3** in CD_3OD at 400 MHz. Integration of contaminant peak indicates compound purity is greater than 96%.
